# A rare clinical image of pachydermoperiostosis (Touraine-Solente-Gole syndrome)

**DOI:** 10.11604/pamj.2025.52.120.49662

**Published:** 2025-11-20

**Authors:** Twinkle Joshi, Janhavi Patharkar

**Affiliations:** 1 Department of Kayachikitsa, Gokul Ayurvedic College, Gokul Global University, Sujanpur, Siddhpur, India

**Keywords:** Pachyderma, periostosis, PDP

## Image in medicine

Pachydermoperiostosis (PDP) is a rare hereditary disorder characterised by digital clubbing, pachyderma, and hyperhidrosis. It accounts for approximately 3%-5% of all cases of hypertrophic osteoarthropathy, with an estimated prevalence of 0.16 per million population worldwide and a marked male predominance. The condition results from mutations in the solute carrier organic anion transporter family member 2A1 (SLCO2A1) or hydroxyprostaglandin dehydrogenase (HPGD) genes, leading to defective prostaglandin E2 (PGE2) metabolism and its accumulation. PGE2 acts as a potent mediator of bone remodelling and dermal fibroblast proliferation, producing the characteristic skeletal and cutaneous manifestations. We report a case of a 15-year-old adolescent female presenting with severe, non-pitting pachyderma over the trunk, deep, irregular folds displaying the classic “elephant-hide” appearance, digital clubbing, and coarse facial features. Facial features were observed clinically; however, photographic documentation was omitted due to the patient's refusal. The diagnosis was based on clinical presentation and a positive family history, consistent with the primary form of pachydermoperiostosis. Genetic testing for SLCO2A1 or HPGD mutations was advised but declined due to financial constraints. Secondary hypertrophic osteoarthropathy and acromegaly were considered as differential diagnoses and excluded based on clinical evaluation and absence of systemic involvement. The patient was managed symptomatically with NSAIDs for arthralgia and systemic retinoids to reduce pachyderma and sebum production. Follow-up demonstrated softening of the skin and improved quality of life. Pachydermoperiostosis remains a diagnostic challenge due to its rarity and overlap with secondary hypertrophic osteoarthropathy. Clinical suspicion supported by characteristic dermatological and family findings is essential for diagnosis. Although disease progression typically stabilises after a decade, residual deformities may persist. Early recognition, symptomatic management and reconstructive procedures can significantly improve patient outcomes.

**Figure 1:**
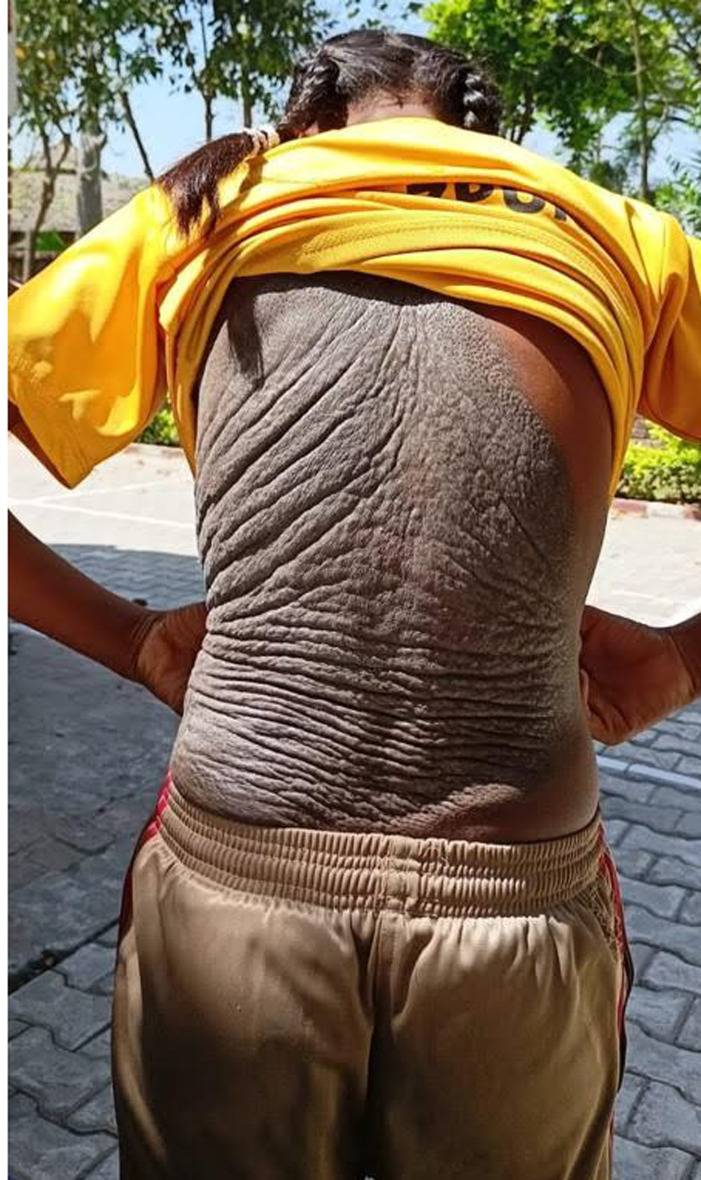
clinical image of pachydermoperiostosis

